# Acute mitral regurgitation following giant coronary artery aneurysm resection: a case report

**DOI:** 10.1186/s13019-026-04167-w

**Published:** 2026-04-19

**Authors:** Akihito Arai, Satoshi Kometani, Kenichiro Takahashi, Akane Kobashi, Ryo Izubuchi, Manato Saito, Minako Hayakawa, Mimiko Tabata

**Affiliations:** 1 Department of Cardiovascular Surgery, Yamato Seiwa Hospital, Kanagawa, Japan; 2 Department of Anesthesiology, Yamato Seiwa Hospital, Kanagawa, Japan; 3 Department of Cardiovascular Surgery, Mayo Clinic, 200 First St. SW, Rochester, MN 55905 USA

**Keywords:** Giant coronary artery aneurysm, Mitral regurgitation, Coronary artery bypass grafting, Left anterior descending artery

## Abstract

**Background:**

Giant coronary artery aneurysms carry a high risk of rupture, thrombosis, and distal embolization. When percutaneous coronary intervention is not feasible in symptomatic cases, surgical resection combined with coronary artery bypass grafting is recommended. However, surgical ligation can interrupt flow to downstream branch arteries and precipitate regional myocardial ischemia.

**Case presentation:**

In a 70-year-old woman with coronary artery disease, coronary angiography showed a 20-mm aneurysm with 90% stenosis of the proximal left anterior descending (LAD) artery, 90% stenosis of the first diagonal branch (D1), and 100% occlusion of the left circumflex (LCx) artery. To resect the aneurysm and perform on-pump beating coronary artery bypass grafting, we used a free gastroepiploic artery graft to the LAD artery and a saphenous vein graft (SVG) from the ascending aorta to the LCx artery, with a piggyback anastomosis between the two grafts. After LAD artery aneurysm resection, acute severe mitral regurgitation and left ventricular dysfunction developed during weaning from cardiopulmonary bypass. Transesophageal echocardiography revealed a posteriorly directed eccentric mitral regurgitant jet with leaflet tethering, suggestive of acute anterolateral papillary muscle dysfunction. Because D1 ischemia was suspected to be contributing to the papillary muscle dysfunction and hemodynamic instability, an additional sequential SVG anastomosis to D1 was created, which resolved the mitral regurgitation and stabilized hemodynamics.

**Conclusions:**

Ligation of a giant proximal LAD aneurysm can markedly alter coronary flow and precipitate unrecognized ischemia, including ischemia affecting the papillary muscle or broader myocardial territories. Because such perfusion dependence cannot be identified reliably on preoperative imaging, surgeons should be prepared to promptly revise the revascularization strategy and consider mechanical circulatory support if hemodynamic instability occurs.

**Supplementary Information:**

The online version contains supplementary material available at 10.1186/s13019-026-04167-w.

## Background

Coronary artery aneurysms are observed in 0.3%–4.9% of patients who undergo coronary angiography (CAG) [1]. Aneurysms, whose diameters exceed four times that of the normal coronary artery or are larger than 8 mm are classified as “giant coronary artery aneurysms” and carry high risks of rupture and embolization [2]. Although asymptomatic or small aneurysms may be managed medically or with percutaneous coronary intervention (PCI), surgical intervention is often the first-line treatment for giant coronary artery aneurysms and for symptomatic cases in which PCI is challenging [1]. However, resection or ligation of an aneurysm may compromise blood flow to coronary branches originating from the aneurysm. This is of particular concern when a single branch supplies the left ventricular papillary muscles [3], as disruption can lead to severe mitral regurgitation (MR) or low cardiac output syndrome.

We describe the case of a 70-year-old woman with a 20-mm giant aneurysm in the proximal left anterior descending (LAD) artery who underwent aneurysm resection and on-pump beating coronary artery bypass grafting (CABG). After the procedure, the patient experienced new episodes of severe MR and left ventricular dysfunction during weaning from cardiopulmonary bypass. This case underscores the importance of promptly reassessing and adapting revascularization strategies when unexpected hemodynamic deterioration occurs after coronary artery aneurysm resection.

## Case presentation

A 70-year-old woman with no remarkable medical history presented with exertional dyspnea. Her family history included sudden cardiac death in her mother and myocardial infarction in her sister. CAG at a local clinic demonstrated triple-vessel disease with a 20-mm giant coronary artery aneurysm in the proximal LAD artery, which prompted referral to our institution.

**Fig. 1:**
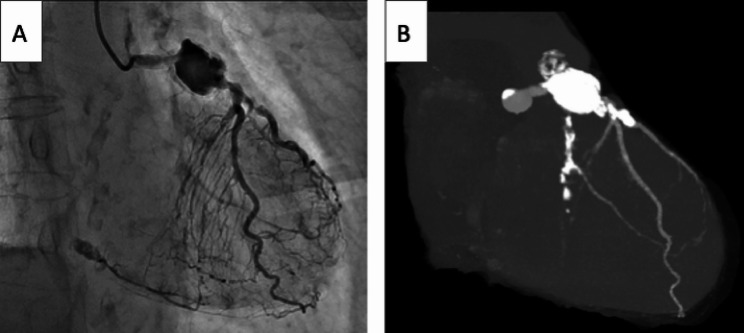
Preoperative imaging. (**A**) Coronary angiography showing a giant aneurysm of the proximal left anterior descending artery. (**B**) Three-dimensional computed tomography reconstruction demonstrating the 20-mm aneurysm and coronary artery anatomy

Physical examination revealed that the patient was hemodynamically stable with no cardiac murmurs. Chest radiography showed a cardiothoracic ratio of 53% and clear lung fields. Electrocardiography depicted T-wave inversions in leads II, III, and V2–V6. Results of routine laboratory tests were unremarkable, except for elevated levels of B-type natriuretic peptide (442 pg/mL). Transthoracic echocardiography depicted an ejection fraction of 55% and hypokinesis of the inferior and posterior walls; mild MR was found on valvular assessment. CAG (Fig. 1A; Video 1) and computed tomography (CT) (Fig. 1B) revealed a 20-mm giant aneurysm with 90% stenosis of the proximal LAD artery.

Additional findings included 90% stenosis at the first diagonal branch (D1), 90% stenosis at the right coronary artery (RCA), and 100% occlusion with severe calcification in the proximal left circumflex (LCx) artery. Because the perfusion territory of D1 was not extensive, we considered bypass grafting to the LAD and LCx to be sufficient without grafting D1. Collateral flow from the septal branches appeared to supply the RCA territory; therefore, bypass grafting to the RCA was not considered necessary.

**Fig. 2:**
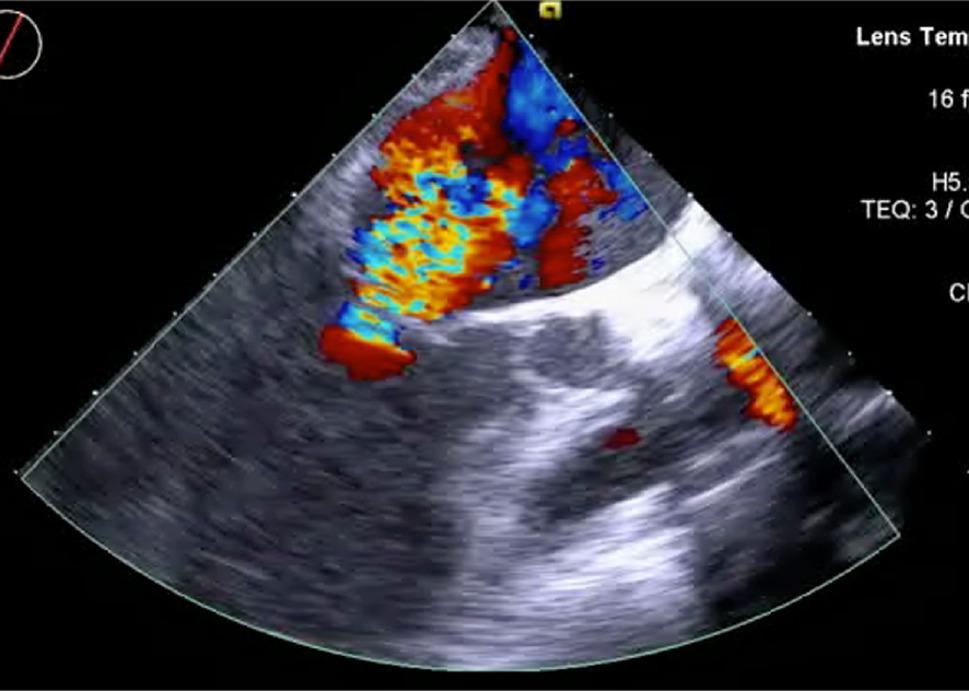
Intraoperative transesophageal echocardiography with color Doppler showing a posteriorly directed eccentric mitral regurgitant jet, suggestive of anterolateral papillary muscle dysfunction

Preoperative transesophageal echocardiography (TEE) confirmed preserved ejection fraction and minimal MR but revealed no additional regional wall motion abnormalities. Under general anesthesia, the patient underwent a median sternotomy. Because flow of the left internal thoracic artery (ITA) was poor due to atherosclerotic calcification and a 10-mm proximal aneurysm was present in the right ITA, both conduits were unsuitable for grafting. We therefore harvested a free gastroepiploic artery (GEA) graft and a saphenous vein (SVG) graft. Cardiopulmonary bypass was established via aortic cannulation and right atrial drainage, while the heart was kept beating. The free GEA graft was anastomosed to the LAD artery, followed by proximal anastomosis of the SVG to the ascending aorta. The proximal end of the free GEA was then anastomosed to the SVG. Transit-time flow measurement of the GEA–LAD graft showed a mean flow of 34 mL/min with a pulsatility index (PI) of 1.6. The giant proximal LAD artery aneurysm was resected, and the proximal and distal ends of the aneurysmal sac were sealed with sutures. Revascularization was completed with a distal anastomosis of the SVG to the obtuse marginal branch (OM). During an attempt to wean the patient from cardiopulmonary bypass, severe MR and global left ventricular dysfunction developed suddenly, which resulted in low cardiac output syndrome (Fig. 2; Video 2). Flow measurement showed that GEA–LAD flow had decreased to 20 mL/min (PI, 1.1) from the initial 34 mL/min, while SVG–OM flow was 46 mL/min (PI, 1.4).

We administered increased levels of catecholamines, but hypotension persisted. TEE revealed a posteriorly directed eccentric regurgitant jet with leaflet tethering, suggestive of acute dysfunction of the anterolateral papillary muscle. Given the possibility that D1 ischemia was contributing to the papillary muscle dysfunction and hemodynamic instability, we created an additional bypass from the aorta via the mid-portion of the SVG to D1. Following D1 revascularization, the total SVG graft flow increased to 166 mL/min (PI, 1.4), and GEA–LAD flow was 28 mL/min (PI, 1.1). Consequently, the MR and left ventricular function improved (Video 2) and thereby allowed successful weaning from cardiopulmonary bypass.

**Fig. 3:**
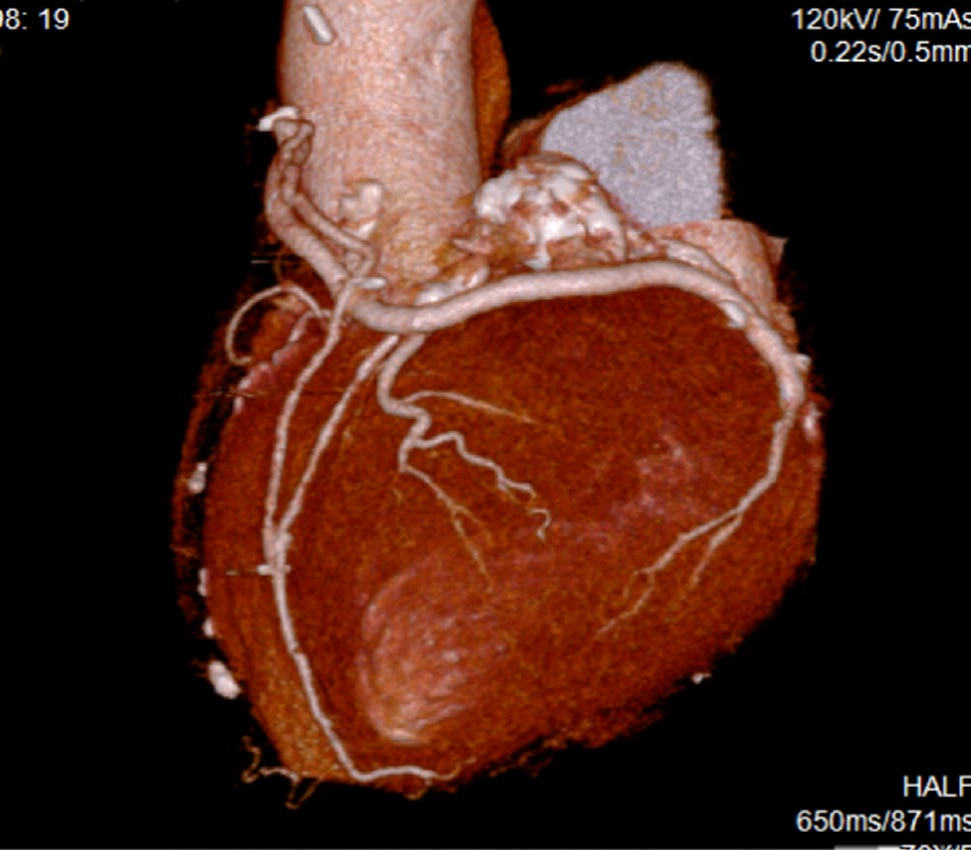
Postoperative three-dimensional computed tomography showing the final graft configuration: saphenous vein graft with piggyback anastomosis to the gastroepiploic artery supplying the left anterior descending artery, with sequential anastomoses to the first diagonal branch and the obtuse marginal artery

Postoperatively, the patient’s hemodynamics remained stable without mechanical circulatory support. She was extubated on postoperative day 1, transferred from the intensive care unit to the general ward on day 5, and discharged on day 22. Postoperative computed tomography confirmed patency of all bypass grafts (Fig. 3).

## Discussion

Appropriate therapeutic intervention is warranted for patients with coronary artery aneurysms, especially those who develop obstructive coronary artery disease [1]. In the present case, the giant aneurysm in the proximal LAD artery alongside calcified stenosis was probably responsible for the patient’s exertional dyspnea. For symptomatic patients in whom PCI is contraindicated, a combination of aneurysm resection or ligation with bypass grafting to adjacent coronary arteries is the preferred treatment [1]. However, aneurysm excision involves occluding the diseased coronary artery; therefore, unrecognized ischemia can occur in branches or distal segments perfused by that artery.

Herein, we created an SVG–GEA piggyback anastomosis to minimize aortic manipulation while maintaining adequate flow in the graft [4]. After ligation of the proximal LAD aneurysm, GEA–LAD flow decreased, and severe MR together with global hypokinesis developed; both resolved following additional bypass grafting to D1.

**Fig. 4:**
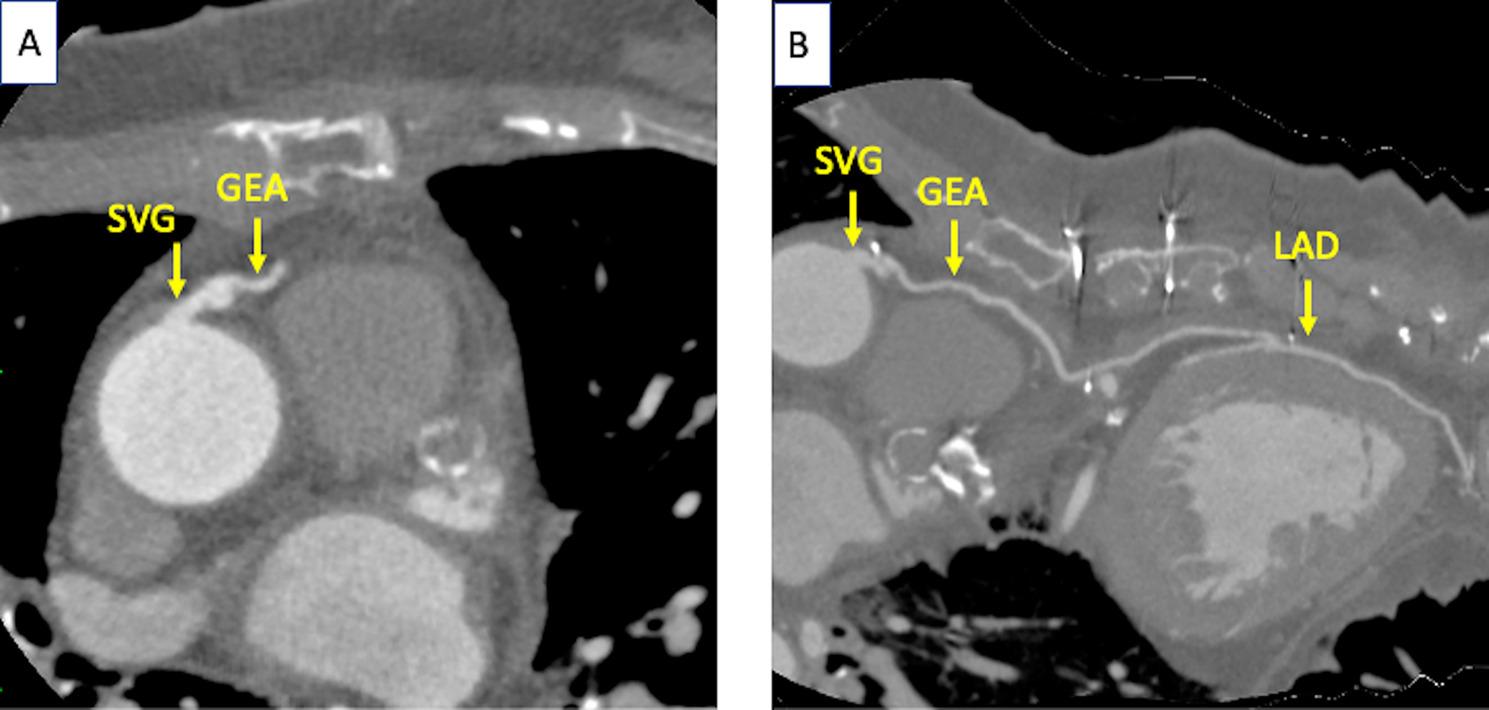
Postoperative contrast-enhanced computed tomography. (**A**) Axial image at the level of the proximal piggyback anastomosis showing patent SVG and GEA grafts without stenosis. (**B**) Curved planar reconstruction along the aorta–SVG–GEA–LAD axis, confirming graft patency throughout its course.GEA, gastroepiploic artery; LAD, left anterior descending artery; SVG, saphenous vein graft

Postoperative computed tomography confirmed patency of both the proximal piggyback anastomosis and the distal GEA–LAD anastomosis without stenosis (Fig. 4A and B), excluding a structural graft problem as the cause of the intraoperative flow reduction, although intraoperative vasospasm of the GEA graft cannot be excluded by postoperative imaging.

Given the absence of a structural cause, two functional mechanisms may explain why D1 revascularization resolved the hemodynamic instability. After proximal LAD ligation, D1 lost its antegrade supply and depended on retrograde flow from the GEA–LAD graft; however, severe ostial stenosis of D1 likely limited this retrograde perfusion. The anterolateral papillary muscle typically receives dual blood supply from diagonal and marginal branches, but in up to 29% of patients its perfusion depends on a single vessel [3]. If D1 was the sole feeding artery, its ischemia after ligation would have directly caused papillary muscle dysfunction and acute MR; D1 revascularization would then have corrected MR, restoring cardiac output and coronary perfusion pressure. Alternatively, even with dual papillary muscle supply, GEA–LAD flow of 20 mL/min may have been insufficient for the combined LAD and collateral-dependent RCA territories; D1 bypass would then have supplemented total myocardial perfusion, improving contractility and cardiac output, thereby raising coronary perfusion pressure and resolving MR. In either scenario, D1 revascularization interrupted the hemodynamic deterioration. This course underscores the importance of accounting for branch dependence preoperatively and of being prepared for unrecognized ischemia that may develop intraoperatively.

Preoperative assessment typically includes coronary CT, which delineates calcification, aneurysm size, and branch anatomy, and CAG, which provides anatomical and functional assessment [5, 6]. However, the microvascular perfusion of critical structures such as the papillary muscles cannot be evaluated accurately before surgery, even when these modalities are combined. The present case highlights how this limitation can lead to clinical complications. Voci et al. [3] attempted to visualize papillary muscle perfusion by injecting albumin microbubbles into a vein graft during CABG and assessing flow with transthoracic echocardiography. However, the routine use of this method is impractical because it requires specialized microbubble preparation, an invasive intraoperative injection, and additional operative time and cost. Therefore, surgeons should be prepared to promptly perform additional bypass grafting or institute mechanical circulatory support if hemodynamic instability occurs intraoperatively.

## Conclusion

In surgery for giant coronary artery aneurysms, ligation of the involved coronary artery may markedly alter coronary flow and precipitate unrecognized ischemia. Accurate preoperative assessment of the blood supply to critical structures such as the papillary muscles remains difficult. Therefore, surgeons should be prepared to promptly revise the revascularization strategy, add grafts, or initiate mechanical circulatory support if hemodynamic instability occurs during the procedure.

## Electronic Supplementary Material

Below is the link to the electronic supplementary material.

Supplementary Material 1—Video 1. Preoperative coronary angiography. Sequential views from multiple angles showing the giant aneurysm of the proximal left anterior descending artery, associated coronary artery lesions, and collateral circulation from septal branches to the right coronary artery territory.

Supplementary Material 2—Video 2. Intraoperative transesophageal echocardiography comparing left ventricular wall motion at three time points: preoperative (left), after aneurysm ligation (center), and after first diagonal branch revascularization (right). Global hypokinesis observed after ligation resolved following D1 bypass grafting.

## Data Availability

The data underlying this article will be shared upon reasonable request from the corresponding author.

## Abbreviations

CABG: Coronary artery bypass grafting
CAG: Coronary angiography
CT: Oomputed tomography
D1: First diagonal branch
GEA: Gastroepiploic artery
ITA: Internal thoracic artery
LAD: Left anterior descending coronary artery
LCx: Left circumflex artery
MR: Mitral regurgitation
OM: Obtuse marginal branch
PCI: Percutaneous coronary intervention
PI: Pulsatility index
RCA: Right coronary artery
SVG: Saphenous vein graft
TEE: Transesophageal echocardiography

## References

[1] Sheikh AS, Hailan A, Kinnaird T, Choudhury A, Smith D. Coronary Artery Aneurysm: Evaluation, Prognosis, and Proposed Treatment Strategies. Heart Views. 2019;20(3):101–8. 31620255 10.4103/HEARTVIEWS.HEARTVIEWS_1_19 PMC6791093

[2] Kato H, Sugimura T, Akagi T, Sato N, Hashino K, Maeno Y, et al. Long-term consequences of Kawasaki disease. A 10- to 21-year follow-up study of 594 patients. Circulation. 1996;94(6):1379–85. 8822996 10.1161/01.cir.94.6.1379

[3] Voci P, Bilotta F, Caretta Q, Mercanti C, Marino B. Papillary muscle perfusion pattern. A hypothesis for ischemic papillary muscle dysfunction. Circulation. 1995;91(6):1714–8. 7882478 10.1161/01.cir.91.6.1714

[4] Baudo M, Cabrucci F, Yakobitis A, Murray C, Torregrossa G. Minimizing stroke risk in off-pump CABG: the role of clampless devices and the piggyback proximal anastomosis technique. Front Cardiovasc Med. 2025;12:1555394. 40099274 10.3389/fcvm.2025.1555394 PMC11912150

[5] Pham V, Hemptinne Q, Grinda JM, Duboc D, Varenne O, Picard F. Giant coronary aneurysms, from diagnosis to treatment: A literature review. Arch Cardiovasc Dis. 2020;113(1):59–69. 31866173 10.1016/j.acvd.2019.10.008

[6] Kawsara A, Nunez Gil IJ, Alqahtani F, Moreland J, Rihal CS, Alkhouli M. Management of Coronary Artery Aneurysms. JACC Cardiovasc Interv. 2018;11(13):1211–23. 29976357 10.1016/j.jcin.2018.02.041

